# Engineered bacterial extracellular vesicles as next-generation precision postbiotics: strategies, challenges and prospects

**DOI:** 10.20517/evcna.2026.09

**Published:** 2026-05-28

**Authors:** Jiali Chen, Qiyan Chen, Baoxian Li, Chunqiang Pan, Roberto Grau, Tao Liu, Shiyu Li, Qingchi Wang, Yanan Wang, Yi Yang, Jintao Cheng, Yuanxiang Jin, Guiling Yang

**Affiliations:** ^1^Xianghu Laboratory, Zhejiang Academy of Agricultural Sciences, Hangzhou 310021, Zhejiang, China.; ^2^College of Biotechnology and Bioengineering, Zhejiang University of Technology, Hangzhou 310032, Zhejiang, China.; ^3^Kyojin S.A., Rosario, Argentina.; ^4^Juventas 4life SL, Madrid 28049, Spain.; ^5^Rawberry Foods Co. Ltd, Huzhou 313000, Zhejiang, China.; ^#^These authors contributed equally to this work.

**Keywords:** Bacterial extracellular vesicles, postbiotics, engineering strategies, biomedical application

## Abstract

Postbiotics, defined as inactivated microorganisms and/or their components conferring a health benefit, represent a paradigm shift from traditional probiotics by offering superior safety, stability, and precise dosing. Bacterial extracellular vesicles (BEVs), natural nanoparticles secreted by bacteria, are key mediators of microbiota-host communication. BEVs have emerged as promising natural postbiotic agents due to their ability to transport bioactive cargo and modulate host physiology. However, the therapeutic application of natural BEVs (nBEVs) is constrained by limitations, including potential safety concerns, lack of target specificity, low yield, and compositional heterogeneity. To overcome these barriers, the concept of engineered BEVs (eBEVs) has been introduced. By employing genetic modification of parent bacteria, surface functionalization, and tailored cargo loading, eBEVs can be precisely designed. This engineering transforms nBEVs into advanced, targeted delivery platforms that significantly expand the scope and efficacy of postbiotics. This review explores the vast potential of eBEVs as next-generation precision postbiotics to treat multiple disorders. Despite the transformative potential, translating eBEVs into clinical practice faces challenges. These include scalable manufacturing processes, comprehensive *in vivo* safety and pharmacokinetic profiling, and the establishment of regulatory frameworks. Future progress hinges on interdisciplinary efforts to develop smart, stimulus-responsive BEVs, enhance endosomal escape efficiency, and conduct rigorous clinical trials. Ultimately, eBEVs stand at the forefront of a new era in microbiota-based therapeutics, merging the biocompatibility of natural vesicles with the precision of synthetic nanomedicine.

## INTRODUCTION

The microbiome refers to the collection of bacteria, fungi, and other microorganisms, identified through metagenomics and meta-transcriptomic analysis, that live in the gut, other human mucosae (e.g., oral, nasal, vaginal mucosae), and on the skin surface. Breakthrough research on the microbiome is opening new avenues for disease treatment^[1]^. While traditional probiotics can exert health benefits by positively modulating the immune system, competitively inhibiting pathogens, enhancing intestinal barrier function, and regulating lipid metabolism^[2]^, their application is significantly limited by the difficulty that microbes face in maintaining viability and colonize the gastrointestinal tract (GI tract), and inconsistent effects^[3]^. As a consequence, the concept of postbiotics has emerged in the microbiome field. According to the definition of the International Scientific Association for Probiotics and Prebiotics (ISAPP), postbiotics are preparations of non-living microorganisms and/or their components that are beneficial to host health^[4]^, which can address the limitations of probiotics. Studies have shown that postbiotics can even be more effective than live bacteria in certain cases, such as inactivated *Akkermansia muciniphila* (*A. muciniphila*), which demonstrates superior performance in increasing GLP-1 levels and improving liver function^[5]^. This transformation marks the shift from relying on the activity of microorganisms for broad-spectrum regulation to an era of “non-living preparations” characterized by precision, stability, and safety.

Extracellular vesicles (EVs) are non-replicating, lipid bilayer-enclosed nanoparticles released by cells, with diameters ranging from 20-400 nm. They carry various biologically active components, including DNA, RNA, lipids, proteins, and metabolites^[6]^. EVs released by bacteria are commonly referred to as bacterial-derived extracellular vesicles (BEVs)^[7]^. They represent one of the key underlying mechanisms behind the detrimental or beneficial effects of many pathogenic, commensal, and probiotic bacteria^[8]^. Among them, those derived from Gram-negative (G-) bacteria are called outer membrane vesicles (OMVs), while those derived from Gram-positive (G+) bacteria are defined as cytoplasmic membrane vesicles (CMVs)^[9]^.

Current research shows that BEVs represent novel potential postbiotic candidates with health benefits^[10]^. BEVs are capable of penetrating intra- and extra-cellular barriers, mediating extensive crosstalk with mammalian cells, and delivering their cargo to the host^[8,11]^. BEVs possess inherent postbiotic properties. They can mediate the interaction between the microbiome and the host, which has attracted widespread attention and is under intensive investigation^[12]^. These are referred to as natural BEVs (nBEVs).

However, the application of nBEVs as postbiotics carries certain risks, as they may contain uncontrolled components like toxins and immunogenic substances, potentially leading to safety concerns^[13]^. BEVs derived from G- bacteria consistently contain lipopolysaccharide (LPS), a pro-inflammatory activator, triggering severe host immune responses^[14-16]^. Some BEVs are associated with pathogenesis and serve as long-distance delivery vehicles for virulence factors related to adhesion, invasion, antimicrobial resistance, and host immune regulation^[17]^. Additionally, due to the inability to fully control and standardize the cultivation conditions, batch-to-match differences of nBEVs lead to heterogeneity^[18]^. The poor targeting ability and low yield of nBEVs also hamper the clinical application^[19,20]^. These uncontrolled components and variability present potential safety challenges for the postbiotic application of nBEVs.

**Figure 1:**
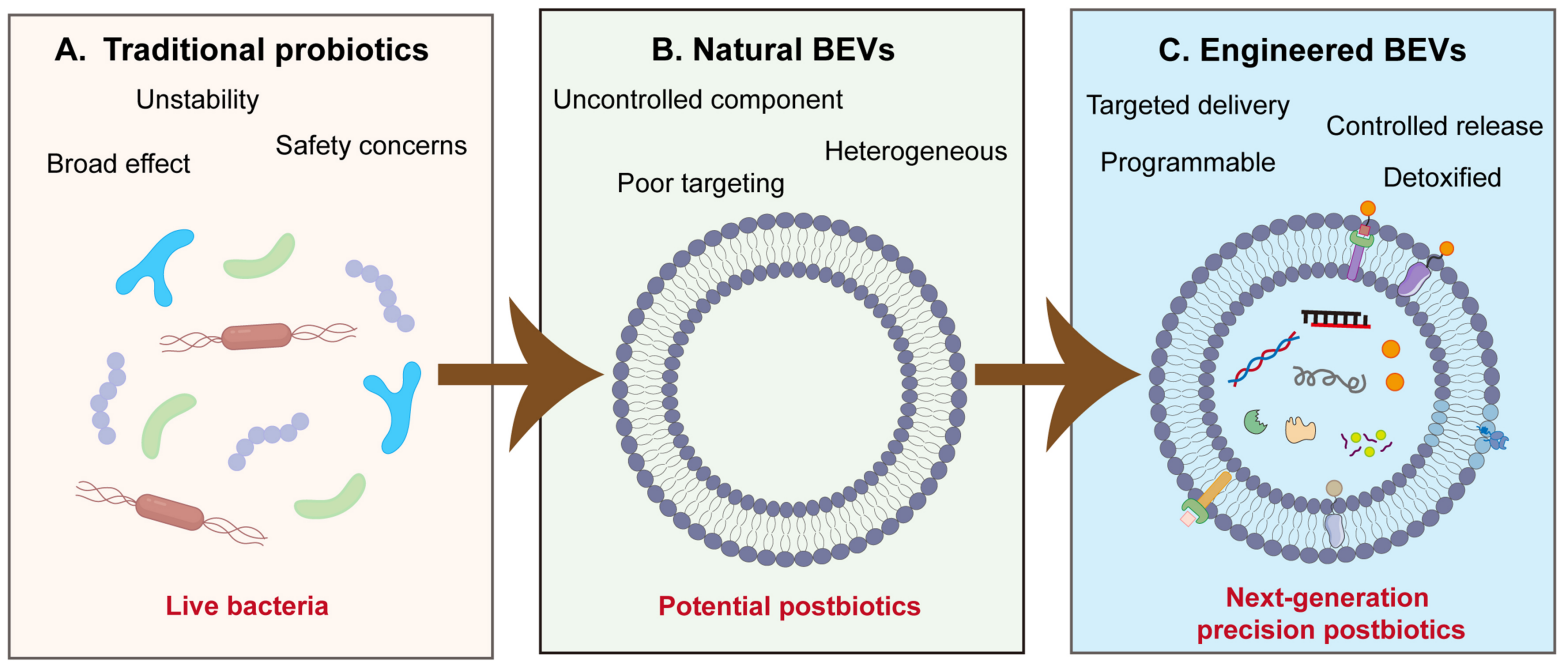
Paradigm shift from traditional probiotics to eBEVs as next-generation precision postbiotics. (A) Traditional probiotics: live beneficial bacteria but with limited stability, potential safety risks, and non-specific broad-spectrum effects; (B) nBEVs: improve general host health but have remaining issues with composition, safety, and stability; (C) eBEVs: precise platform with targeted delivery, controlled release, programmable payload loading, and detoxified profiles. BEVs: Bacterial extracellular vesicles; nBEVs: natural bacterial extracellular vesicles; eBEVs: engineered bacterial extracellular vesicles.

In order to better utilize BEVs, enhancing security, improving efficacy and new functions, and achieving precise delivery, diverse engineering approaches can be employed to modify nBEVs. Main approaches include (i) modifying toxin genes; (ii) controlling the expression of exogenous proteins; (iii) surface modification of proteins or peptides for targeting purposes; (iv) physical methods such as membrane fusion, ultrasound, and squeezing wrapping, combining with nanoparticles; and (v) regulating genes involved in BEV synthesis to enhance BEV yields^[21-23]^. Unlike traditional probiotics and postbiotics, engineered BEVs (eBEVs) are engineered to achieve four distinct levels of precision: (i) specific targeted delivery via surface engineering; (ii) controlled and stimuli-responsive cargo release; (iii) programmable therapeutic payloads tailored to specific diseases; (iv) reduction of off-target toxicity through detoxification. Also, the therapeutic effects of traditional probiotics and postbiotics are difficult to fully predict or standardize because of the complex and undefined components of bacteria. In contrast, eBEVs are welldefined nanoscale vesicles that emerge as postbiotics of the next generation, which can be precisely engineered at the genetic, physical, and chemical levels, while retaining natural biocompatibility and intrinsic bioactivity. Collectively, eBEVs, as next-generation precision postbiotics, offer a level of control that cannot be achieved by unmodified nBEVs or other probiotics/postbiotic formulations [Figure 1]. This review will systematically elaborate on the strategies to design and construct eBEVs, their application prospects in various diseases, and the challenges in their clinical translation.

## OVERVIEW OF nBEVs

G+ bacteria have thick cell walls rich in peptidoglycan (PG) and teichoic/lipoteichoic acids (TA/LTA), whereas G- bacteria have inner and outer membrane structures rich in LPS, lack of TA/LTA, and a lower content of PG. Both types of bacteria can spontaneously form and secrete BEVs^[24]^; however, the formation mechanisms differ due to the different cell structures. G- bacteria mainly secrete BEVs through two pathways: the one pathway produces OMVs that contain only the components of the outer membrane. The other pathway, resulting from cell wall degradation leads to explosive outer membrane vesicles (EOMVs) or outer-inner membrane vesicles (OIMVs) that contain inner and outer membranes as well as cytoplasmic components of the^[9,16]^. G+ bacteria, due to their thicker PG layer, were previously believed to be unable to secrete BEVs. However, not until 2009 were G+ bacteria discovered to produce vesicles through a process similar to “bubble-like cell death”^[25]^. The specific mechanism is still unclear and may be related to the action of autolysins or phagocytic lysis enzymes^[26]^.

Oral administration of BEVs and their absorption through the intestines is the most prevalent route. BEVs carry numerous miRNAs, proteins, and lipids from parental cells, enabling them to traverse the intestinal barrier, transfer bioactive molecules into intestinal epithelial cells, and effectively diffuse into the circulatory system for transmission to peripheral organ systems^[27,28]^. BEVs administrated via surface routes require the ability to penetrate bodily barriers and reach host cells. Within 24 h, BEVs from *Lactobacillus paracasei* can reach the deepest part of the dermis and alleviate tumor necrosis factor-α (TNF-α)-induced skin inflammation^[29]^. Moreover, BEVs can enter respiratory epithelial cells and release their contents to stimulate lung immune cells while inducing protective immune responses^[30]^.

**Figure 2:**
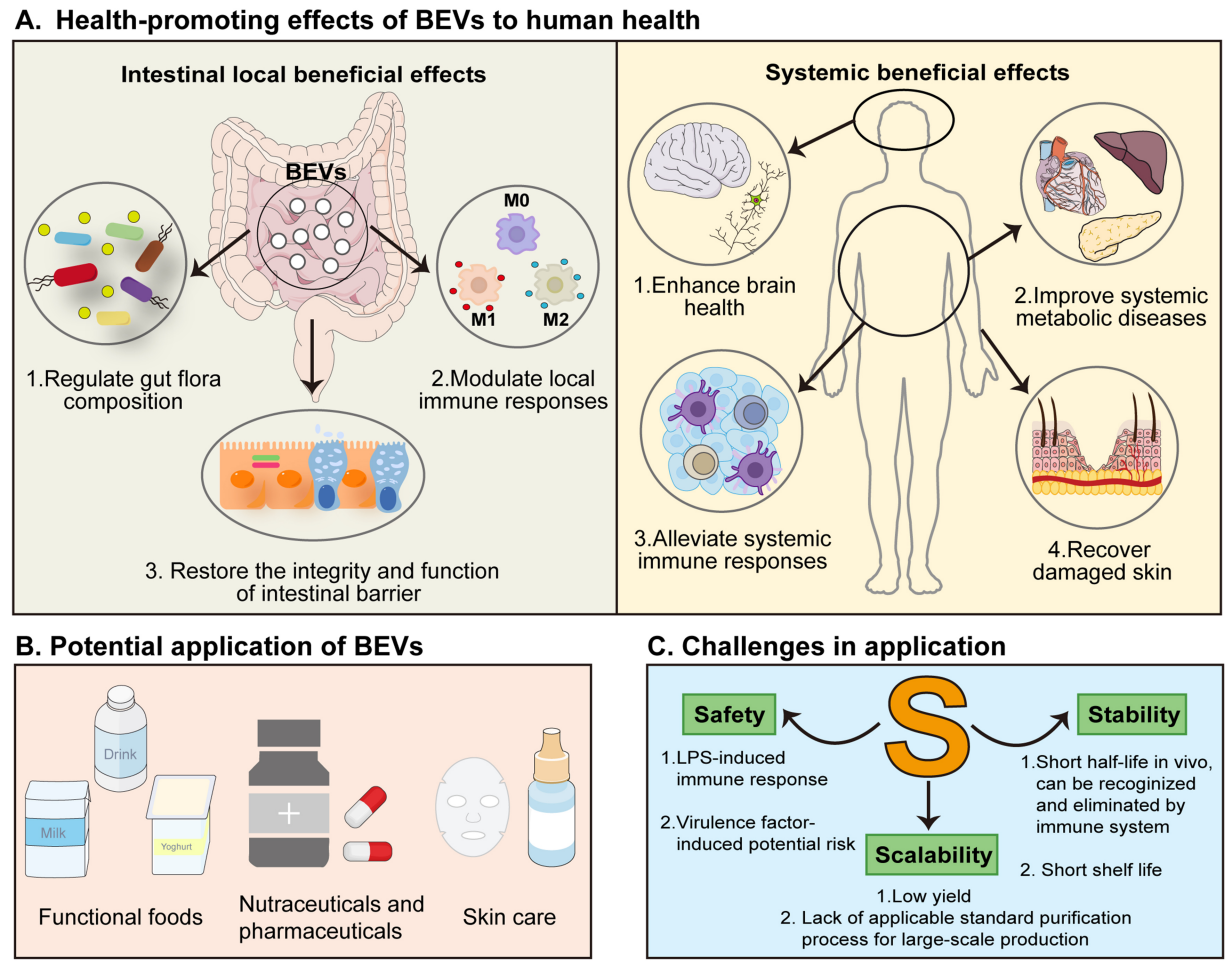
Health-promoting effects of nBEVs and potential challenges in their applications. (A) BEVs exert their health-promoting effects on the intestinal tract by regulating the composition of the host’s intestinal microbiota, modulating local immune responses in the intestine, and restoring the integrity and function of the intestinal barrier. Furthermore, BEVs also have a positive impact on the overall health of the host (including the brain, metabolic diseases, immune system, and skin); (B) Potential applications of BEVs as postbiotics in the food and biomedical industries; (C) Current challenges for the safe and effective clinical application of BEVs. BEVs: Bacterial extracellular vesicles; nBEVs: natural bacterial extracellular vesicles; LPS: lipopolysaccharide.

BEVs possess a wide range of functions, contingent upon their unique nanostructure and the diverse biomolecules they carry. To date, BEVs have been confirmed to exert broad health-promoting effects [Figure 2 and Table 1]. A large body of research has explored the beneficial effects of BEVs in local intestinal environment, including enhancing the intestinal barrier integrity^[45]^, modulating the gut microbiota^[46,47]^, and regulating local inflammatory responses^[48]^. In addition, BEVs exhibited systemic health effects, such as improving brain health^[49,50]^, regulating anti-tumor immune responses^[51,52]^, and alleviating metabolic diseases^[33,34]^. These studies indicate that nBEVs are potentially vital regulators of general health and may provide protective effects against diseases. However, issues such as safety, stability, and scalability require further refinement.

**Table 1:** nBEVs derived from different bacterium and their therapeutic effects

**Bacterium**	**Model**	**Mechanism**	**Effects**	**Ref.**
L*ysobacter enzymogenes* C3	*F. subglutinans* Infection		Deliver antifungal compounds to treat fungal diseases	[31]
*Bacteroides longum* KACC 91563	Diarrhoea due to food allergy		Induce apoptosis of BMMCs，prevention of food allergy	[32]
*A.muciniphila MucT* ATCC BAA-835	HFD induced diabetic mice		Increased tight junction protein expression in Caco-2 cells and ultimately improved intestinal barrier integrity	[33]
*A. MucT* ATCC BAA-835	HFD induced fat mice		Improved the intestinal barrier integrity, inflammation, energy balance, and blood lipid profile and glucose level	[34]
*Escherichia coli* Nissle 1917	DSS induced mouse colitis	↓ Body weight loss ↓ Disease activity index	Reduced DSS-induced weight loss and ameliorated clinical symptoms and histological scores	[35]
*Lactobacillus animalis* ATCC 35046	GC-induced ONFH		Enhancing blood vessel abundance, maintaining osteogenic activity, reducing cell apoptosis, and attenuating trabecular bone and marrow damage, thus protecting the mice from GC-induced ONFH	[36]
*A.muciniphila*	DSS-induced colitis	↓ IL-6	Protected DSS-induced IBD phenotypes, such as body weight loss, colon length, and inflammatory cell infiltration of colon wall	[37]
*Bacteroides fragilis*	TNBS-induced experimental colitis		Induce immunomodulatory effects and prevent experimental colitis	[38]
*Clostridium butyricum*	DSS-induced colitis in mice	Inhibition of NF-kB pathway activation Improving the intestinal barrier	Alleviate bacterial dysbiosis，improve intestinal barrier integrity and inhibit the inflammatory response	[39]
*A.muciniphila*	HFD/CCL4-induced liver injury		Improvement in gut health Anti-inflammatory effects on liver and adipose tissues	[40]
LGG	ALD	Mediated by intestinal AhR signaling ↑ Intestinal interleukin‐22‐Reg3 ↑ Nrf2-tight junction signaling pathways	Protected the intestine from alcohol‐induced barrier dysfunction and the liver from steatosis and injury	[41]
*Lactobacillus paracasei*	TNF-α-induced inflammatory phenotypes in human skin		Restored the TNF-α-induced epidermal malformation, abnormal proliferation of keratinocytes in the basal layer, and reduction in dermal collagen synthesis	[29]
*Lactobacillus plantarum*	Skin aging	Modulated the mRNA expression of ECM related genes	Reduction in skin moisture content and increase skin density Suppressed wrinkle formation and pigmentation	[42]
*Lactobacillus plantarum* ZS62	Alcohol-induced subacute liver injury	Reducing inflammation and enhancing antioxidant capacity	Attenuated alcohol-induced weight loss; Prevented morphological changes in hepatocytes; Reduced AST, ALT, Haase, PC III and inflammatory cytokines; Down-regulated inflammation-related genes and up-regulated lipid- and oxidative-metabolism genes	[43]
*Lactobacillus plantarum* DP189	MPTP-induced PD mice	Activated the expression of Nrf2/ARE and PGC-1α pathways and suppressed the NLRP3 inflammasome	Increased the levels of SOD, GSH-Px, IL-10, decreased the levels of MDA, ROS, TNF-α, IL-6 and IL-1β. Reduced the α-SYN accumulation in SN. Reshaped the gut microbiota in PD mice	[44]

↓Downregulation; ↑upregulation. nBEVs: natural bacterial extracellular vesicles; *F. subglutinans*: *Fusarium subglutinans*; BMMCs: bone marrow mononuclear cells; *A. muciniphila*: *Akkermansia muciniphila*; HFD: high-fat diet; Caco-2: human epithelial colorectal adenocarcinoma cell line; DSS: dextran sulfate sodium; GC: glucocorticoid; ONFH: osteonecrosis of the femoral head; IL-6: interleukin-6; IBD: inflammatory bowel disease; TNBS: trinitrobenzenesulfonic acid; NF-κB: nuclear factor kappa-B; CCl4: carbon tetrachloride; LGG: *Lactobacillus rhamnosus* GG; ALD: alcoholic liver disease; AhR: aryl hydrocarbon receptor; Nrf2: nuclear factor erythroid 2-related factor 2; TNF-α: tumor necrosis factor-α; mRNA: messenger RNA; ECM: extracellular matrix; AST: aspartate aminotransferase; ALT: alanine aminotransferase; PC III: procollagen type III; MPTP: 1-methyl-4-phenyl-1,2,3,6-tetrahydropyridine; PD: Parkinson’s disease; ARE: antioxidant response element; PGC-1α: peroxisome proliferator-activated receptor gamma coactivator-1α; NLRP3: NOD-like receptor family pyrin domain containing 3; SOD: superoxide dismutase; GSH-Px: glutathione peroxidase; IL-10: interleukin-10; MDA: malondialdehyde; ROS: reactive oxygen species; IL-1β: interleukin-1β; α-SYN: α-synuclein; SN: substantia nigra.

## ENGINEERING STRATEGIES FOR BEVs

To meet the clinical application requirements, multiple engineering strategies have been developed to modify nBEVs with enhanced yield, therapeutic efficacy, stability and targeting capacity. The modification methods for nBEVs engineering can be mainly classified into three core aspects, focusing on increasing the production of BEVs, modifying the membrane surface, and remolding of inner lumen^[18]^. This review thoroughly discusses the advanced approaches for BEVs engineering to address the current application bottlenecks.

### Enhancing BEVs yield

**Figure 3:**
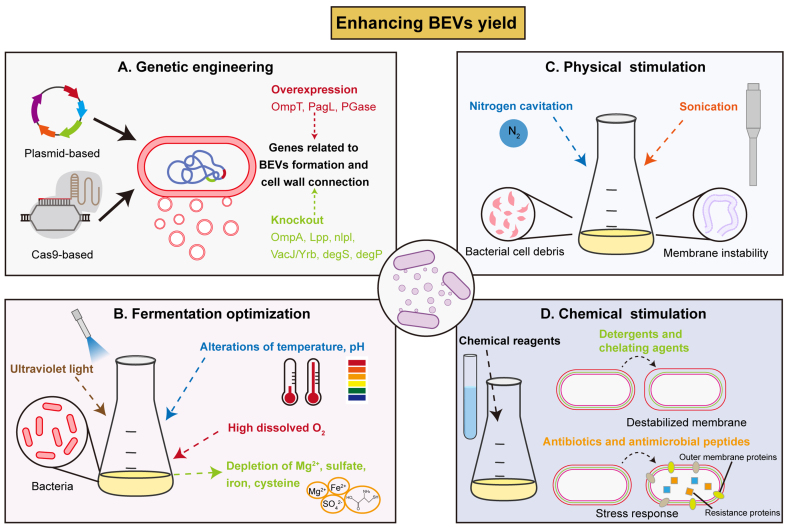
Strategies to enhance BEVs yield. (A) Utilization of genetic engineering techniques (recombinant plasmids, CRISPR-Cas9) to overexpress or knockout genes related to BEVs formation and cell wall composition; (B) Fermentation optimization, such as UV light stimulation, temperature/ pH alteration, and depletion of nutrients to enhance BEVs yield; (C) Physical stimulation with extreme environmental forces (nitrogen cavitation, sonication) to produce cell debris and induce membrane instability, thus enhance BEVs production; (D) Chemical reagents treatment (detergents, chelating agents, antibiotics) to induce more BEVs production as a self-protective response. BEVs: Bacterial extracellular vesicles; OmpT: outer membrane protease T; PagL: lipid A 3-O-deacylase PagL; PGase: peptidoglycan hydrolase; OmpA: outer membrane protein A; Lpp: murein lipoprotein; NlpI: lipoprotein NlpI; DegP: periplasmic serine endoprotease DegP; CRISPR-Cas9: clustered regularly interspaced short palindromic repeats-associated protein 9; UV: ultraviolet.

Current research mainly focuses on enhancing the production of BEVs by genetically engineering of the parent strains, optimizing cultivation conditions and downstream processes [Figure 3]. Utilizing the various strategies presented below to increase BEVs yield could pave the way for their application.

#### Genetic modification

Genetic engineering modification utilizes gene editing technology to regulate the expression of genes related to the biogenesis of BEVs to achieve large-scale generation of BEVs^[53]^. The formation of BEVs in G- bacteria (in which most studies have been performed) requires the detachment of the outer membrane from the PG layer and inner membrane, thus breaking the connections between the layers of the cell wall (i.e., inner membrane-PG-outer membrane structures) facilitates the release of BEVs^[54]^.

The gene overexpression strategy is explored to enhance BEVs production. The outer membrane protease OmpT of enterohemorrhagic *Escherichia coli* (*E. coli*) (EHEC) influences the bacterium’s ability to adapt to the host environment by dynamically regulating the production, composition, and size of OMVs. Overexpression of the *ompT* significantly increases OMVs production with a 40-fold increase compared with the wild type and further modulates bacterial growth^[55]^. Another study reveals that lipid A (a component of the LPS) deacylation is a vital mechanism driving OMVs biogenesis. Overexpression of deacylase PagL in *Salmonella typhimurium* induces membrane morphological changes, leading to outer membrane bending and promoting the formation of OMVs^[56]^. PG constitutes a common component of the cell wall in both G+/G- bacteria^[57]^. Up-regulating the expression of PG hydrolase PGase enhances PG hydrolysis, thereby inducing the BEVs secretion. This universal strategy has been validated in both G+/G- bacteria. An autonomous PGase expression system yields a 9.37-fold increase in Escherichia coli Nissle 1917 (EcN)-derived BEVs^[58]^. Furthermore, a combined strategy integrating membrane curvature engineering with dynamic PGase modulation results in a remarkable 149-fold increase in vesicle production^[59]^.

Aside from gene up-regulating modification, another widely utilized genetic engineering method is the knockout/inhibition of genes associated with BEVs formation. This strategy targets the molecules related to crosslinking of the three layers (outer membrane, PG, and inner membrane) of G- bacteria^[53,54]^. Knockout of OmpA in *Acinetobacter baumannii*^[60]^ and lipoprotein (Lpp) in *E. coli*^[61]^, as well as the deletion of nlpI in EcN^[62,63]^, all result in the disruption of the integrity and stability of the cell wall and lead to enhanced BEVs release. Additionally, the accumulation of LPS and PG precursors in the cell wall further exacerbates the envelope stress response, thereby promoting augmented BEVs secretion^[64]^. For instance, silencing genes associated with the *VacJ*/*Yrb* transport system leads to the accumulation of phospholipids in the outer membrane and induces the BEVs generation, resulting in 3.9-fold and 4.3-fold increases in yield, respectively, for *VacJ* and *Yrb* gene deletions, while vesicle sizes remain similar^[65,66]^. The deletion of genes encoding proteins that suppress BEVs formation or degrade misfolded proteins, such as *degS* and *degP* protease-coding genes, causes misfolded proteins to accumulate in the periplasm, which increases membrane stress, triggering enhanced OMV production^[64]^.

Enhancing the production of BEVs through genetic engineering offers a key advantage in achieving fundamental breakthroughs in yield while simultaneously endowing BEVs with new functionalities. However, this strategy also faces significant challenges, including potential growth impairment in modified cells, technical complexity, and stringent regulatory approval requirements when used as pharmaceutical products. Overall, it represents a cutting-edge strategy with immense potential but demanding technical and human safety requirements.

#### Fermentation optimization

By altering the culture conditions and applying environmental insults, the general stress response of bacteria is induced, thereby increasing the release of BEVs as a protective response^[67]^. Fermentation optimization mainly involves altering certain nutrients and cultivation parameters.

The lack of Mg^2+^ increases the protein level of PagL, which catalyzes the deacetylation of lipid A on LPS, and causes membrane bending and release of BEVs, with 3-5-fold increase^[67]^. The depletion of sulfate and iron from the culture medium can decrease the expression of *VacJ*/*Yrb* transporter and lead to phospholipid accumulation, which disrupts the membrane symmetry^[65,68]^. Also, in *Neisseria meningitidis*, exhaustion of cysteine and high dissolved oxygen levels cause oxidative stress, which triggers hypervesiculation^[69,70]^.

Besides the change in culture medium components, extreme environmental factors (temperature, pH, ultraviolet light) can also influence the generation of BEVs. In *Pseudomonas*, when cultivated at elevated temperature, the combined effects of increased membrane fluidity and enhanced interaction of the LPS with *Pseudomonas* quinolone signal (PQS) synergistically promotes the biogenesis of BEVs^[71]^. Similarly, during the formation of BEVs in *Serratia marcescens*, low temperatures of 22 or 30 ℃ induce stress response, leading to the production of large quantities of BEVs^[72]^. When faced with extreme pH condition and ultraviolet (UV) radiation, the bacteria can develop self-protective mechanisms and generate more BEVs^[73,74]^. However, the alteration of the culture conditions typically results in BEVs heterogeneity; thus, culture parameter optimization must be combined with rigorous quality assessment.

#### Physical and chemical stimulation

To enhance BEVs production, two additional strategies can be employed: physical modification and chemical treatment. These methods effectively promote vesicle formation and release by altering the physiological state of bacteria or their membrane stability.

Physical treatment primarily induces BEVs formation through mechanical force. Sonication can enhance BEVs generation by increasing the membrane instability; however, the complete protein repertoire may not be fully retained in the resulting BEVs^[75,76]^. Nitrogen cavitation generates a mechanical force within a chamber that rapidly disrupts cell membranes, subsequently releasing intracellular contents. The cell membranes spontaneously form nanoscale compartments, producing membrane-derived nanovesicles composed entirely of the original cell membrane, thereby achieving rapid BEVs preparation (up to 20-to 30-fold relative to native secretion) and better preserving protein integrity than sonication^[77]^.

Chemical methods directly target bacterial cell membranes with chemical reagents or induce stress responses to promote BEVs generation. Detergents, such as deoxycholate and sodium dodecyl sulfate, effectively remove LPS from BEVs and destabilize cell membranes to stimulate BEVs secretion while reducing the unintended innate immune response triggered by LPS^[56,75,78]^. Chelating agents such as Ethylenediaminetetraacetic Acid (EDTA) cause mild membrane instability by neutralizing the negative charge of LPS, an effect that can preserve LPS and natural cargo in BEVs^[79-81]^. Moreover, exposure to sublethal concentrations of antibiotics (such as ciprofloxacin) can activate protective stress responses in bacteria, promoting BEVs release with 2-to 4-fold enhancement by temporarily altering membrane state^[82-84]^. Antimicrobial peptides exhibit antibiotic-like effects in inducing BEVs release, but the generated vesicles typically contain higher levels of phosphatidylglycerol, potentially compromising their thermal stability^[85,86]^.

Notably, basal BEV yield varies substantially among bacterial strains. Genetic backgrounds, especially envelope stress response systems and PG network rigidity, can directly influence both native vesiculation levels and the fold‑increase achievable by hypervesiculating mutations, as well as the final product quality. Therefore, strain selection is a prerequisite for achieving target yield and cargo integrity for any given therapeutic application. Moreover, the choice of yield enhancement strategy has notable implications for BEVs quality and scalability. While genetic engineering offers the highest yield improvements and high batch-to-batch consistency, engineered strains may alter size distribution and cargo composition. Regarding scalability, genetically engineered strains are well-suited for industrial fermentation, as the modified bacteria can be cultured at large scale using standard bioreactors. In contrast, physical methods require specialized equipment and are more challenging to scale up, while chemical induction offers simplicity but raises concerns about residual reagent contamination. Therefore, a comprehensive evaluation of the characteristics of the resulting BEVs must be conducted based on specific application objectives.

### Engineering the membrane of BEVs

**Figure 4:**
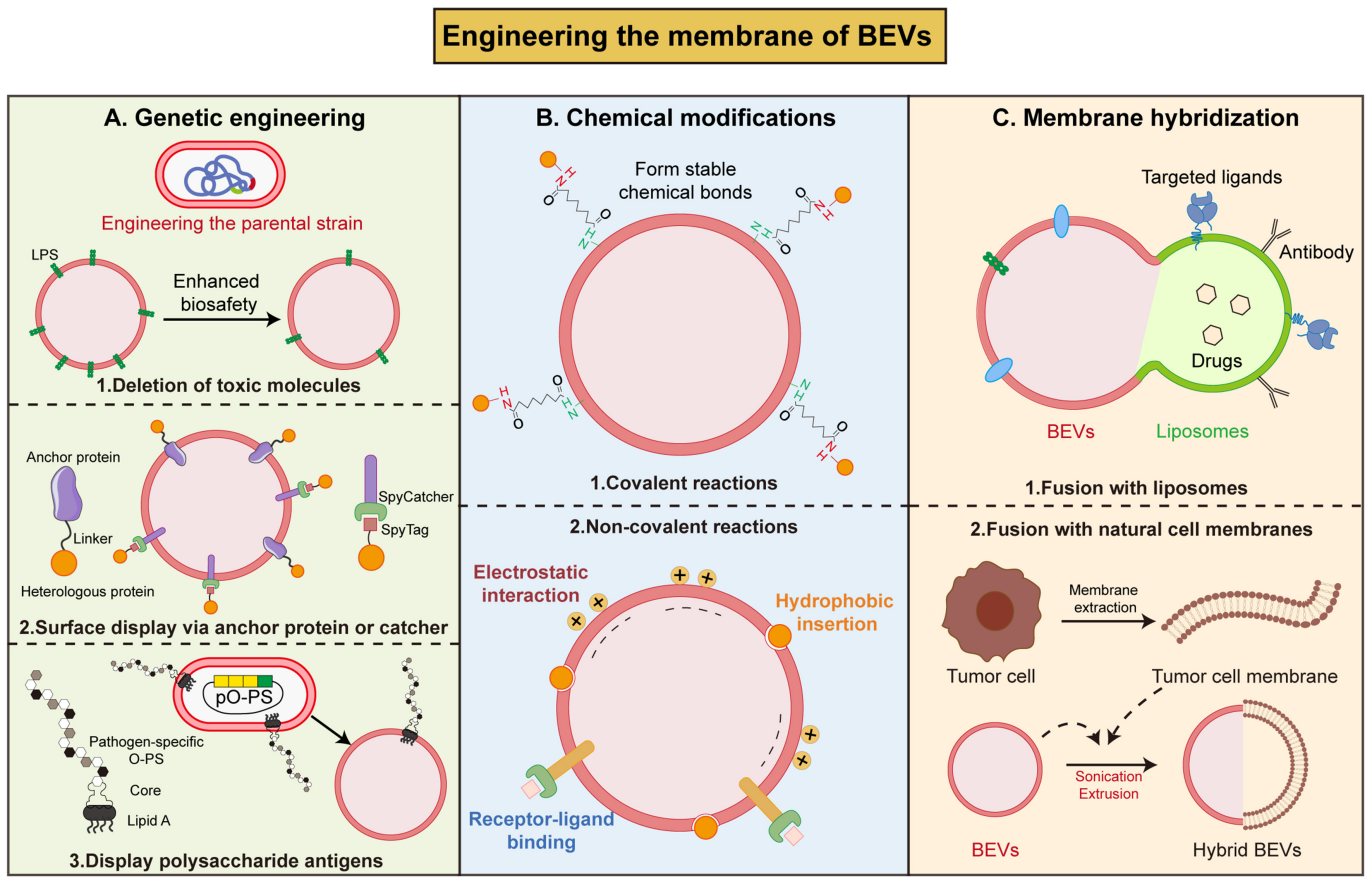
Strategies to engineer the membrane surface of BEVs. (A) Utilizing genetic engineering techniques to eliminate toxic molecules in BEVs membrane surface, fuse desired proteins with anchor protein or catcher, or displaying polysaccharide antigens on BEVs surface; (B) Using covalent (forming stable chemical bonds) or non-covalent (electrostatic interaction, hydrophobic insertion, receptor-ligand binding) reactions to coat antigens on BEVs surface; (C) Fusion with liposomes or natural cell membranes forms hybrid BEVs. BEVs: Bacterial extracellular vesicles; LPS: lipopolysaccharide; SpyTag: SpyTag peptide; SpyCatcher: SpyCatcher protein; O-PS: O-polysaccharide (O-antigen); PO-PS: pathogen-specific O-polysaccharide.

Modifying the membrane surface of BEVs endows them with enhanced targeting and delivery capabilities, improves biosafety and regulates immunogenicity, and optimizes pharmacokinetics and stability. Membrane surface modification methods are primarily categorized into three types: genetic engineering, chemical modifications, and membrane hybridization [Figure 4].

#### Genetic engineering

Genetic manipulation of parental bacteria at the molecular level enables the deletion of toxic molecules during BEVs formation or the natural expression of desired proteins on the surface during BEVs biosynthesis. Both G^+^ and G^–^ bacteria can produce molecules with toxic or potentially toxic effects that play important roles in bacterial infections^[87]^. Knocking out or mutating selected genes in the LPS, TA and LTA synthetic pathways can fundamentally alter the membrane composition of BEVs, in a way that reduces their toxicity^[53]^. For example, eliminating bacterial virulence genes (*msbB*) at source via *CRISPR*/*Cas9* gene editing can enhance the biosafety of BEVs^[88]^.

In addition to the engineering of BEVs membrane composition to reduce toxicity, genetic engineering techniques have also been employed to display exogenous proteins on BEVs surface. Through surface display technology, desired functional proteins or peptides are fused with the native membrane proteins of BEVs, enabling the protein to be efficiently and stably anchored to the membrane^[89]^. Currently, the most common carrier proteins include bacterial cytolysin A (ClyA), OmpA, Omp W and β-lactamases (Bla)^[90-92]^. ClyA is a pore-forming toxin capable of traversing the bacterial inner membrane to localize to the outer membrane^[93]^. By fusing exogenous proteins to the C-terminal of ClyA, these proteins can be directly localized to the membrane surface and to the lumen of OMVs^[94,95]^. Genetic engineering of BEVs by surface display technology is widely used to enhance the immunogenicity^[53]^. It was reported that the fusion of the coding region of the programmed death-ligand 1 (PD-L1) with ClyA could yield engineered OMVs carrying PD-L1. This genetic modification of OMVs enhances their accumulation at tumor site, and induces blockade of death-ligand 1, resulting in a more effective anti-tumor immune response^[96]^. Moreover, the fusion of ClyA with cell-penetrating peptide (CPP) achieves an augmented tumor-targeted binding and penetration^[97]^, and fusion with hyaluronidase (HAase) degrades the extracellular matrix (ECM) within the tumor microenvironment, thereby enhancing the delivery efficiency of therapeutic drugs^[98]^. Beyond the ClyA system, autotransporter-based platforms have also been developed for antigen display on membrane surfaces. The hemoglobin protease (Hbp) autotransporter from *E. coli* has been engineered into a versatile surface display carrier that can be expressed at high density on OMVs. By fusing heterologous antigens to Hbp, multiple *Mycobacterium tuberculosis* antigens (ESAT6, Ag85B, and Rv2660c) were successfully displayed on the surface of OMVs, eliciting antigen-specific immune response^[99]^. As for G+ bacteria (*Bacillus subtilis*, *B. subtilis*), Chen *et al*. have constructed a peptide display platform by fusing spore coat protein CotC with antimicrobial peptide Cathelicidin-BF (Cathe). The BEVs secreted by this engineered strain contains Cathe and demonstrates antimicrobial activity and further alleviates dextran sulfate sodium (DSS)-induced colitis^[100]^.

Apart from protein/peptide components, utilizing glycoengineering strategies, polysaccharide antigens can be inserted on the BEVs surface to generate glycoengineered BEVs as vaccine candidates^[101]^. Price *et al*. developed a modular plasmid containing crucial genes for enzymes of *S. pneumoniae* serotype 14 capsule (CPS14) synthesis. When this plasmid was transformed into O-antigen-deficient *E. coli*, the synthesized capsular polysaccharide could bind to the lipid A core of LPS, thereby enabling the generation of eBEVs carrying the CPS14 antigen to prevent multiple infections^[102]^. Similarly, expression of tularemia O antigen polysaccharide (O-PS) in O-antigen-deficient *E. coli* enables the assembly of glycosylated BEVs with protective immunogenic activity via the core glycosylation of LPS^[103]^.

The core advantages of utilizing genetic engineering for BEVs membrane modification lie in its precision and *in situ* nature. By modifying the parental bacterial genome, the modifying components can be displayed *in situ*, covalently and at high density on the membrane during BEVs biosynthesis. Simultaneously, this strategy fundamentally alters BEVs components, thereby enhancing biosafety. However, the technique is complex and time-consuming, potentially disrupting bacterial physiology and yield.

#### Chemical modifications

Employing biochemical reactions, exogenous functional molecules are attached to the surface of BEVs, endowing them with enhanced targeting capabilities and therapeutic potential. Chemical methods are mainly categorized into covalent and non-covalent binding.

Covalent reaction anchors exogenous molecules to the membrane surface via the formation of stable chemical bonds, including conjugation, click chemistry, and aldehyde amine condensation to modify BEVs^[104]^. Forming amide bonds between the carboxyl groups present on the surface of BEVs and the amino groups of 3-aminophenylboronic acid (PBA) allows the attachment of PBA to the surface of BEVs. The chemically modified BEVs possesses the ability to target sialic acids present on the surface of tumor cells. Furthermore, HAase expression combined with PBA surface modification increased deep tumor penetration of OMVs by 266.7% and enhanced small interfering RNA (siRNA) silencing efficiency by 70.5%^[105]^. In addition, the use of cross-linking agents such as thiol-maleimide, adipic acid, or carbodiimide makes it possible to covalently attach protein antigens, inhibitors or nanobodies to BEVs^[106,107]^. To streamline the process, researchers have developed improved methods based on bis(sulfosuccinimidyl)suberate cross-linking or reductive amination, enabling direct coupling via lysine residues on the antigen without requiring pre-derivatization^[107,108]^. This feature renders the Generalized Modules for Membrane Antigen (GMMA) system an efficient “plug-and-play” vaccine development platform.

Non-covalent modifications rely on diverse physical interactions and specific-binding, offering a flexible and efficient alternative approach for the functionalization of BEVs^[104]^. Firstly, molecular attachment can be realized through multivalent electrostatic interactions or hydrophobic insertion. Sun *et al*. modified Angiopep-2 and Trans-Activator of Transcription (TAT) peptide onto the surface of BEVs membrane via lipid insertion, enabling the encapsulation of a chemotherapy drug^[88]^. The SpyCatcher-SpyTag protein conjugation system enables efficient, modular antigen display on BEVs surfaces by forming specific isopeptide bonds with SpyTag peptides on target proteins^[109]^. Furthermore, through receptor-ligand binding, RGD peptide binds to integrin α_v_β_3_ for the loading of indocyanine green^[110]^. These non-covalent approaches collectively broaden the applications of BEVs in drug delivery and immunotherapy.

Chemical modification offers diverse strategies for functionalizing BEVs: covalent modifications provide stable, durable linkages suitable for vaccine development and targeted therapies; non-covalent modifications offer greater flexibility for drug delivery, molecular display, and rapid functionalization. Collectively, these approaches advance the application prospects of BEVs in drug delivery, immunotherapy, and diagnostics.

#### Membrane hybridization

Through physical forces (extrusion, ultrasonication, freeze-thaw cycles), BEVs can be fused with functional liposomes or cell membranes to form hybrid vesicles that combine the characteristics of both. The emerging EVs-liposome fusion strategy has developed into a powerful approach to overcome the limitations of EVs in application, and expand the functional capabilities of EVs^[111]^. As liposomes are artificially synthesized, their surfaces can be modified to achieve targeted, prolonged, and sustained release, while possessing advantages as high biocompatibility and low immunogenicity^[112]^. Zhai *et al*. engineered hybrid vesicles by fusing OMVs derived from attenuated *Salmonella* with photothermal sensitive liposomes, demonstrating significant antitumor efficacy^[113]^. Also, Pan *et al*. used modified liposomes loaded with luteolin and borneol to fuse with OMVs isolated from *Stenotrophomonas maltophilia* using an extrusion technique. The constructed hybrid OMVs-liposomes achieve deep mucosal penetration capability and retention capacity within the inflamed colon^[114]^. Integrating liposomes technology with BEVs produces innovative nano-pharmaceuticals that simultaneously exploit the immunogenicity of BEVs and the precise targeting and tunable drug release profiles of liposomes, paving the way for a versatile platform in targeted therapy.

Another strategy for BEVs membrane hybridization is the fusion with natural cell membranes, especially eukaryotic membranes. Abundant studies have reported hybrid membrane-based nanovesicles by combining bacterial OMVs with tumor cell membranes to confer the unique advantages of BEVs as personalized cancer vaccines in situations where neoantigens are not readily accessible^[115]^. Specifically, Wang *et al*. reported a hybrid membrane platform by fusing OMVs with B16-F10 tumor cell membrane to utilize both the advantages of OMVs and tumor cells to achieve better antitumor efficacy^[116]^. In another work, researchers showed the successful construction of a heterologous fusion membrane tumor vaccine by fusing BEVs from *Staphylococcus aureus* with B16-F10 cell membrane to activate immune responses and prevent tumor development^[117]^. Wang *et al*. constructed hybrid nanovesicles by fusing OMVs secreted by *E. coli* with membranes extracted from tumor-homing macrophages to selectively target tumor tissues and inhibit tumor growth^[118]^. Additionally, extensive research has also been conducted to obtain hybrid BEVs from other bacteria. Firstly, *A. muciniphila*, *Bifidobacterium longum* (*B. longum*), and *Bifidobacterium breve* (*B. breve*) were treated with lysozyme to prepare protoplasts. Then, hybrid vesicles were synthesized via ultrasonication and extrusion, which demonstrated enhanced targeting capacity towards tumors and peripheral lymphoid organs^[119]^.

In summary, engineering modifications to the surface of BEVs represent a key strategy for optimizing the *in vivo* behavior, enhancing therapeutic efficacy, and improving safety. The aforementioned approaches may be employed individually or in combination, thereby enabling the bespoke design of ideal BEV delivery vehicles tailored to diverse clinical application scenarios. This transformation is the core step in advancing BEVs from natural messengers to precision medicine tools.

### Engineering the lumen of BEVs

**Figure 5:**
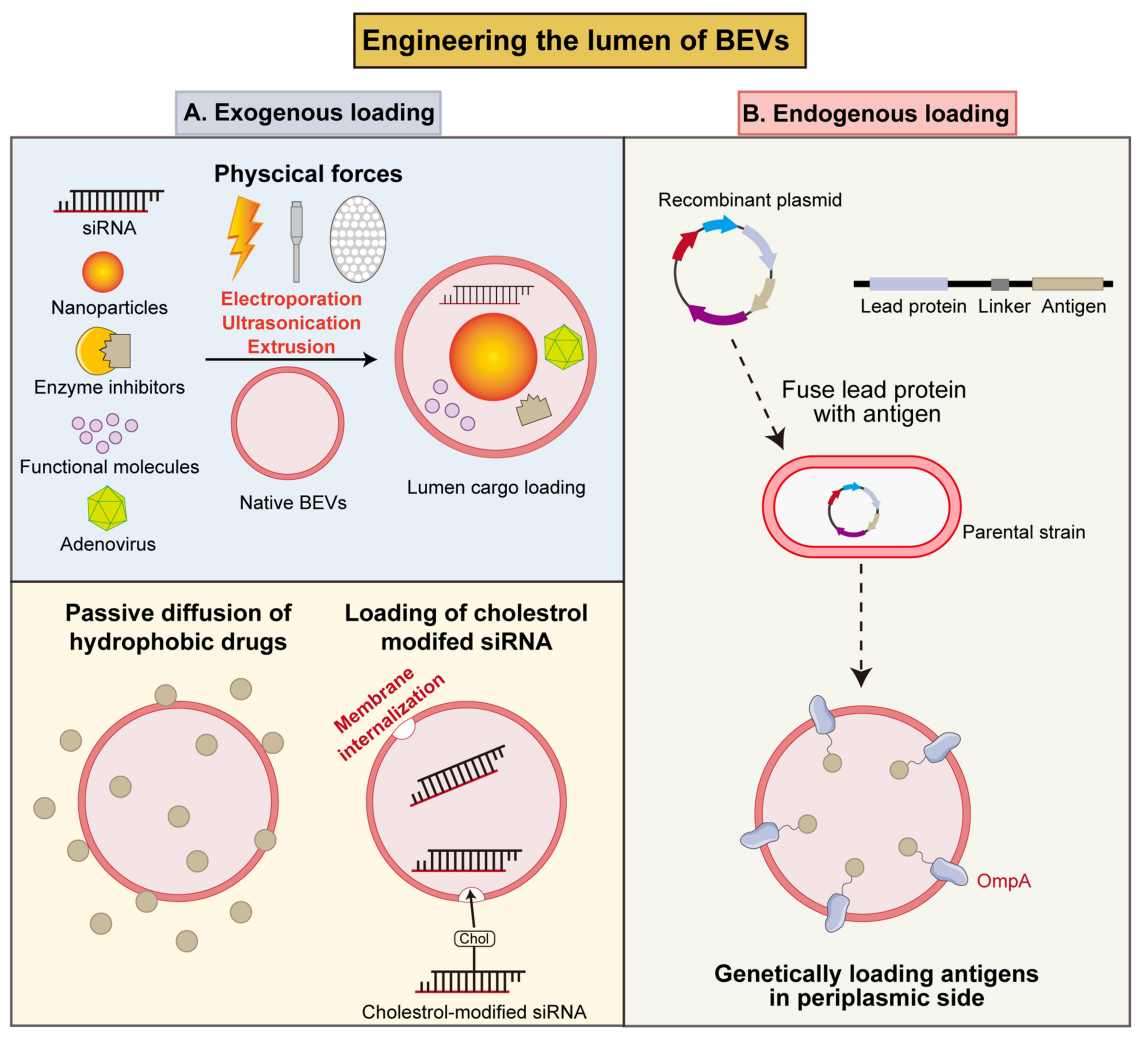
Strategies to engineer the lumen of BEVs. (A) Exogenous loading of cargos by physical forces (electroporation, ultrasonication and extrusion), passive diffusion, or cholesterol modification into BEVs lumen; (B) Endogenous loading of antigens into the BEVs lumen through genetic engineering strategy. BEVs: Bacterial extracellular vesicles; siRNA: small interfering RNA; OmpA: outer membrane protein A.

By using exogenous (physical and chemical methods) and endogenous (genetic engineering) strategies, therapeutic cargos are loaded into the lumen of BEVs, thereby further enhancing the functions of BEVs [Figure 5].

#### Exogenous loading

Firstly, physical treatments could achieve the loading of exogenous cargos in the inner cavity of BEVs. Electroporation has been used to load small gold nanoparticles (AuNPs) in *Pseudomonas aeruginosa*-derived BEVs via the pores in the lipid bilayer generated by a transient electric field^[120]^. Also, electroporation successfully loads siRNA and specific inhibitors in BEVs to regulate tumor microenvironment and further inhibit the tumor growth^[121,122]^. Another study utilized ultrasonic bath to load fucoxanthin (Fx) in *Lactobacillus plantarum* (*L. plantarum*) derived synthetic membrane vesicles to treat colitis^[123]^. Ban *et al*. encapsulated oncolytic adenovirus (Ads) in *E. coli* derived BEVs through extrusion to enhance the intra-tumoral accumulation of Ads and achieve autophagy-cascade-augmented immunotherapy^[124]^.

Furthermore, chemical approaches represent another prospective nanotechnology for the loading of therapeutic molecules. A prevalent strategy for antibiotic loading into the BEVs lumen is passive loading, which is based on co-culturing bacteria in media containing sublethal concentration of antibiotics, which facilitates the intrinsic incorporation of antibiotics during the natural biogenesis of BEVs^[125]^. Additionally, cholesterol-modified siRNA can be loaded into BEVs via hydrophobic interactions^[105,126]^. This mild, non-invasive encapsulation strategy not only achieves efficient siRNA loading, but also preserves the integrity and biological activity of both BEVs and the cargos.

#### Endogenous loading

As for the development of BEVs vaccine, it is crucial to ensure the high immunogenicity of BEVs^[127]^; this requires antigen-presenting cells (APCs) to engulf antigen-containing particles. Thus, engineering the parental bacteria to load more antigens offers a method for enhancing immunogenicity by localizing bioactive molecules within the periplasmic space^[53]^. Multiple antigens were successfully encapsulated into the lumen of BEVs, thereby inducing protective immune responses against pathogen infection. For instance, *Chlamydia muridarum*/*Chlamydia trachomatis* serovar D (DO) DO serine protease HtrA antigen can induce severe humoral and cellular immune responses *in vivo*. In one report, HtrA was fused with OmpA and expressed in *E. coli* to deliver it to the lumen of BEVs. The obtained eBEVs effectively stimulate functional immune responses^[128]^. Similarly, Fantappiè *et al*. selected a group of heterologous antigens (*GAS* Slo, SpyCEP, Spy0269 and *GBS* SAM_1372) to fuse with OmpA. The antigens incorporated into the BEVs lumen induced the production of high antibody titers in mice^[129]^. Collectively, these results indicate that BEVs carrying internalized antigens undergo structural alterations upon interaction with immune cells, leading to antigen exposure or release, which is then recognized by ACPs to mount a specific antibody response. Thus, the simplicity of genetic engineering paves the way for diverse strategies to load antigens into BEVs.

### Comparative summary of engineering strategies

The engineering strategies discussed above can be broadly categorized into three main technologies: genetic engineering, physical methods, and chemical approaches, each with distinct advantages, limitations, and application scenarios.

Genetic engineering enables precise yield enhancement, surface display, and LPS detoxification with high scalability but faces technical complexity and regulatory hurdles. Physical methods (sonication, cavitation, extrusion) are rapid and broadly applicable but risk cargo damage and batch inconsistency, issues that need to be solved. Chemical approaches offer operational simplicity and versatile functionalization but may compromise BEV integrity or leave residues. The optimal strategy depends on the application, bacterial strain, and regulatory pathway.

The choice of engineering strategy ultimately depends on the intended application, the bacterial strain involved, and the regulatory pathway. Often, hybrid strategies combining two or more approaches yield the most clinically promising BEV platforms. Table 2 provides a systematic comparison of these strategies, highlighting their relative strengths and limitations, as well as the typical applications.

**Table 2:** Comprehensive comparison of different BEVs engineering strategies

**Strategies**	**Specific approaches**	**Advantages**	**Disadvantages**	**Applications**	**Ref.**
Genetic engineering	Gene knockout/overexpression (*nlpl, ompA, lpp, ompT, pagL, degS, degP*)	1. Significant increase in BEVs yield; 2. Uniform and stable BEVs composition; 3. Suitable for large-scale production; 4. Endow BEVs with inherent functions	1. High technical requirements; 2. Time-consuming; 3. May affect the growth and viability of the engineered strain	Mass production of standardized BEVs; Vaccine development	[55,56,60-64]
	Surface display system (ClyA, OmpA, Lpp-OmpA)	1. *In situ*, covalent, high-density display; 2. Excellent stability	1. Fusion proteins may affect the function of anchor proteins; 2. Strain-dependent efficiency	Targeted ligand presentation; Vaccine antigen delivery	[89-98]
	LPS/Lipid A modification (*msbB*, *lpxL*, *lpxM*, *pagL*, *pagP*)	1. Reduce endotoxin toxicity at source; 2. Preserve natural adjuvant activity	1. Excessive attenuation may reduce the immunostimulatory effect; 2. The strategies for modifying different bacteria vary	Therapeutic applications with high safety requirements; Development of vaccine adjuvants	[88]
Physical methods	Ultrasonication, nitrogen cavitation, extrusion	1. Rapid and straightforward; 2. Do not require genetic manipulation; 3. Applicable to multiple strains	1. May compromise the natural structure of the cargo; 2. Poor batch-to-batch consistency; 3. Risk of contamination by impurities; 4. High dependence on equipment	Rapid preparation of BEVs; Strains that are difficult to genetically modify; Preparation of hybrid vesicles	[75-77,124]
	Membrane fusion	1. Combine the immunogenicity of BEVs with the targeted/controlled-release properties of synthetic carriers; 2. High degree of functional designability	1. Unstable fusion efficiency; 2. High heterogeneity of fusion products; 3. Complex quality control	Targeted drug delivery; Personalized cancer vaccines	[113-118]
Chemical approaches	Surface covalent modification (amide bonds, click chemistry, cross-linking agents)	1. Stable and long-lasting connections; 2. Controllable level of functionality; 3. High “plug-and-play” flexibility	1. Affect the function of natural proteins on the surface of BEVs; 2. Residual reagents pose a safety risk	Vaccine development (GMMA platform); Targeted ligand conjugation	[104-108]
	Surface non-covalent modification (electrostatic adsorption, hydrophobic insertion, ligand-receptor binding)	1. Gentle operation and simple conditions; 2. Does not damage the structure of BEVs; 3. High functionalization efficiency	1. The stability of the modification is relatively poor	Rapid functionalization screening; Development of drug delivery carriers	[88,105,109,110]
	Chemically induced hypersecretion (detergents, EDTA, antibiotics)	1. Easy to operate and cost-effective; 2. Significant increase in yield; 3. Simultaneously reduces LPS content	1. Residual reagents may compromise safety; 2. Alter the composition and immunogenicity of BEVs; 3. Poor batch-to-batch consistency	Rapid expansion of laboratory capacity; Preparation of attenuated BEVs	[56,75,78-84]

BEVs: Bacterial extracellular vesicles; nlpI: new lipoprotein I; OmpA: outer membrane protein A; Lpp: Braun’s lipoprotein; OmpT: outer membrane protease VII; PagL: lipid A 3-O-deacylase; DegS: periplasmic serine endoprotease DegS; DegP: periplasmic serine endoprotease DegP; ClyA: cytolysin A; LPS: lipopolysaccharide; Lipid A: lipid A domain of lipopolysaccharide; msbB: lipid A biosynthesis myristoyl transferase; lpxL: lauroyl acyltransferase; lpxM: myristoyl acyltransferase; GMMA: generalized modules for membrane antigens; EDTA: ethylenediaminetetraacetic acid.

## THERAPEUTIC APPLICATION OF eBEVs

**Figure 6:**
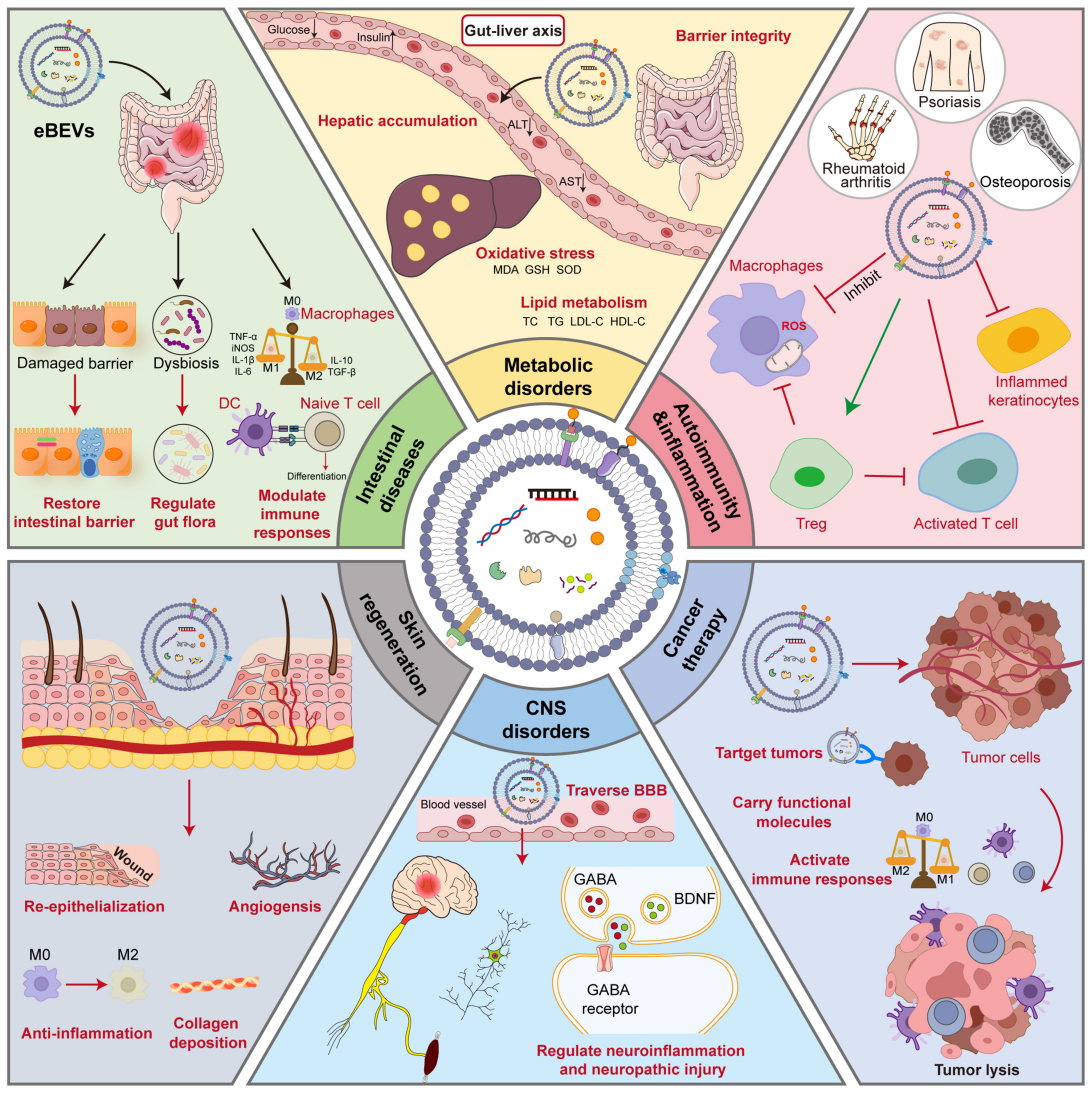
Application of eBEVs in treating various diseases. eBEVs could participate in disease regulation through multiple pathways and targets, covering aspects such as intestinal barrier repair, immune regulation, metabolic intervention, neuroprotection, and anti-tumor activity. Firstly, eBEVs can act on damaged intestinal barriers by restoring their integrity and regulating the balance of the microbiota. In metabolic disorders, eBEVs participate in regulating oxidative stress indicators (such as MDA, GSH, SOD) and lipid metabolism-related indicators (TC, TG, LDL-C, HDL-C), thereby intervening in metabolic disorders. In autoimmune diseases such as psoriasis, rheumatoid arthritis, and osteoporosis, eBEVs demonstrate regulatory potential. eBEVs can regulate the differentiation of naive T cells, affect immune responses and inflammasome activity, promote the function of Treg, and regulate the transformation of macrophages from M1 type to M2 type, exerting anti-inflammatory effects. In tumor treatment, eBEVs can carry functional molecules, activate immune responses, and directly promote tumor cell lysis, demonstrating targeted therapeutic potential. For central nervous system diseases, eBEVs can cross the blood-brain barrier and regulate GABA, BDNF, and related receptor pathways, thereby alleviating neuroinflammation and nerve damage. eBEVs can also promote skin regeneration by promoting re-epithelialization, vascular regeneration, and collagen deposition. This figure reflects the diversity and synergy of eBEVs as multifunctional nanocarrier, highlighting the integrated mechanism in barrier repair, immune remodeling, metabolic regulation, neuroprotection, and tumor intervention. eBEVs: Engineered extracellular vesicles; BEVs: bioengineered extracellular vesicles; ALT: alanine aminotransferase; AST: aspartate aminotransferase; MDA: malondialdehyde; GSH: glutathione; SOD: superoxide dismutase; TC: total cholesterol; TG: triglycerides; LDL-C: low-density lipoprotein cholesterol; HDL-C: high-density lipoprotein cholesterol; ROS: reactive oxygen species; TNF-α: tumor necrosis factor-α; iNOS: inducible nitric oxide synthase; IL-1β: interleukin-1β; IL-6: interleukin-6; TGF-β: transforming growth factor-β; DC: dendritic cell; Treg: regulatory T cell; BBB: blood-brain barrier; CNS: central nervous system; GABA: γ-aminobutyric acid; BDNF: brain-derived neurotrophic factor.

Leveraging the versatile engineering strategies described above, eBEVs have been developed into tailored therapeutic platforms for a wide range of diseases. Compared with nBEVs, eBEVs offer enhanced targeting accuracy, improved cargo delivery efficiency, and tunable immunomodulatory properties. To date, preclinical studies have demonstrated the promising potential of eBEVs in treating intestinal disorders, metabolic diseases, autoimmune conditions, cancers, central nervous system (CNS) disorders, and skin injuries. In this section, we systematically review the application of eBEVs across these disease categories [Figure 6].

### Intestinal diseases

nBEVs, acting as pivotal mediators of microbe-host interactions, can regulate intestinal immune homeostasis and barrier function through synergistic effects. The mechanism can be summarized in the following aspects; first, nBEVs can directly enhance the integrity of the intestinal epithelial barrier. For instance, *Roseburia intestinalis*-derived BEVs are reported to upregulate the expression of tight junction proteins, thereby reducing intestinal epithelial permeability and thus collaborating with the host to resist pathogen invasion^[130]^. Similarly, *L. plantarum* and its BEVs can also promote mucus secretion to preserve and repair the gut barrier^[131]^. Secondly, nBEVs can modulate the phenotypic polarization of innate immune cells, particularly macrophages. BEVs derived from *L. plantarum* and *L. johnsonii* can induce macrophages to polarize towards the anti-inflammatory M2 phenotype^[131,132]^. In addition, nBEVs can reshape the gut microbiota composition by increasing the abundance of beneficial bacteria and suppressing the abundance of potential pathogenic bacteria, thus restoring the microbial balance^[47]^. Collectively, nBEVs constitute a multidimensional therapeutic network through a synergistic mechanism of “barrier repair-immunomodulation-microbiome remodeling”, offering a potential intervention strategy for gastrointestinal diseases like colitis.

eBEVs, as an emerging nanotherapeutic platform, demonstrate enormous potential in the treatment of intestinal diseases owing to their inherent biocompatibility, superior mucosal penetration capabilities, and amenability to engineering modification. Through targeted modification and functional enhancement of nBEVs, the resulting eBEVs demonstrates high targeting precision and synergistic therapeutic effects. Because the production of nBEVs is typically low, researchers have genetically engineered *E. coli* and *L. plantarum* to obtain engineered strains with enhanced capacity of BEVs production. Moreover, these high-yield eBEVs have significantly improved the response to DSS-induced colitis in various aspects, suggesting eBEVs hold promise as a therapeutic product requiring no live bacteria^[59,133]^. Also, eBEVs can serve as efficient and safe targeted delivery vehicles for specific molecules or miRNA^[123,134,135]^. For example, genetic engineering technique has been employed to deliver antimicrobial peptide into BEVs of *B. subtilis* via surface display technology. In a DSS-induced acute colitis mouse model, these *B. subtilis*-derived BEVs significantly suppress inflammatory responses, restore the compromised intestinal barrier, and positively modulate the gut microbiota^[100]^. Furthermore, Liang *et al*. employed a physical method to encapsulate Fx within probiotic membrane vesicles derived from *L. plantarum* to construct fucoxanthin-membrane vecisles (FX-MVs). FX-MVs can enhance Fx’s gastrointestinal stability, mitigate colonic inflammatory responses, reshape the gut microbiota, and increase the abundance of short-chain fatty acids^[123]^. The integration with biomaterials to construct local sustained-release systems represents another crucial strategy. Embedding BEVs within thermosensitive or pH-responsive hydrogels enables targeted, sustained release within inflamed colonic regions, maintaining therapeutic concentrations locally while preventing rapid clearance^[136]^. Given the global burden of *Helicobacter pylori* (*H. pylori*) infection and the challenges in vaccine development, recent studies have explored OMV-based strategies. Notably, Liu *et al*. developed a novel recombinant OMV vaccine candidate by knocking down three key LPS modification genes (*lpxE*, *lpxF* and *futB*) in *H. pylori*. These engineered OMVs, loaded with major antigens UreB, VacA and CagA, induced specific humoral immune responses and a Th1/Th2/Th17 mixed response (with Th17 predominant), and markedly protected mice from *H. pylori* infection^[137]^. In an earlier study, the same group showed that recombinant OMVs delivering eukaryotic expression plasmids of cytokines [interleukin-17A (IL-17A) or interferon-γ (IFN-γ)] could act as enhanced adjuvants, inducing stronger humoral and mucosal immune responses than wild-type OMVs or cholera toxin adjuvant^[138]^. Collectively, these findings highlight the potential of eBEVs as both antigen delivery vehicles and safe adjuvants for *H. pylori* vaccine development.

Engineered modifications systematically address the limitations of nBEVs in drug loading capacity, targeting precision, and controllable efficacy through multidimensional strategies. These intelligent eBEVs systems not only amplify their inherent capabilities to repair the gut barrier and modulate the microbiota composition, but also integrate precise drug delivery with active immune modulation in intestinal diseases.

### Metabolic disorders

Metabolic disorders, such as type 2 diabetes mellitus (T2DM), obesity, metabolic-associated fatty liver disease (MAFLD), and hyperuricemia, have become critical global public health challenges^[139-141]^. Conventional drug therapies exhibit limitations in targeting specificity, bioavailability, and multi-target regulation. Recently, EVs have emerged as novel nanocarriers, demonstrating significant potential in the intervention of metabolic disorders due to their inherent biocompatibility, low immunogenicity, and exceptional ability to penetrate biological barriers^[142]^.

Mammalian EVs have demonstrated therapeutic potential. For example, human umbilical cord mesenchymal stem cell-derived EVs enriched with Exendin-4 directly improve glycemic control and renal function in diabetic nephropathy^[143]^. Besides, plant-derived exosome-like nanovesicles (PELNs) from various sources also ameliorate metabolic disorders through multiple pathways. Pomegranate-derived exosome-like nanovesicles (naturally rich in ellagic acid) are reported to restore intestinal barrier function and reduce liver oxidative stress and fibrosis markers in MAFLD model^[144]^. Ginger-derived exosome-like nanoparticles can activate the Phosphatidylinositol 3-kinase/Protein Kinase B (PI3K/Akt) signaling pathway in the liver via their carried miRNAs, which further enhances insulin sensitivity, inhibits gluconeogenesis, and produces hypoglycemic effects in T2DM mice^[145]^. Similarly, the bitter melon-derived EVs loaded with curcumin demonstrate unique targeted delivery capabilities, enabling specific transport of active components to the pancreas via the lymphatic system to restore insulin secretion function^[146]^. Despite the remarkable therapeutic efficacy of these naturally sourced EVs, their complex active constituents and inconsistent batch-to-batch variability have hindered their applications, which has propelled the field towards the development of eBEVs.

The core concept of eBEVs involves transforming probiotics and other GRAS (Generally Recognized As Safe) microorganisms into “living factories” that continuously produce and load therapeutically active proteins into BEVs. This approach addresses the global challenge of delivering macromolecular biological agents (such as enzymes and peptides) via oral administration. The utilization of microfluidics-assisted ultrasound technology allows the encapsulation of Fx within *Lactobacillus*-derived EVs (LCEV), forming LCEV@Fx. BEVs encapsulation prolongs Fx retention time within the GI tract, enhances absorption across the entire serosal layer, and increases its hepatic accumulation. LCEV@Fx reduced white fat accumulation and ameliorated hepatic steatosis in high-fat diet mice by promoting fatty acid oxidation via the AMP-activated protein kinase/Sirtuins 1 (AMPK/SIRT1) pathway and downregulating lipid synthesis-related proteins^[147]^. In another landmark study, researchers genetically engineered probiotics to produce high yields of OMVs, enabling the targeted therapeutic protein (uric acid oxidase) to self-assemble within the OMVs with an encapsulation rate as high as 97.9%. OMV encapsulation provides effective protection for unstable protein drugs, allowing them to withstand gastric acid and digestive enzymes while efficiently traversing the intestinal epithelial barrier into systemic circulation. In hyperuricemic model, oral administration of such eBEVs resulted in continuous clearance of uric acid^[63]^. These techniques are adaptable to multiple therapeutic proteases, providing a universal platform for intervention in a range of metabolic disorders.

The intervention of metabolic diseases with BEVs is in its early stages. Although BEVs protect protein cargo from gastric acid and proteases, efficient intestinal absorption remains challenging. BEVs must traverse the mucus layer, avoid entrapment by intestinal epithelial glycocalyx, and undergo transcytosis to reach systemic circulation. Protein release may occur prematurely in endosomes, reducing bioavailability. Furthermore, protein loading efficiency and maintaining native protein folding during encapsulation are additional hurdles. Future engineering should focus on muco-adhesive surface modifications and endosomal escape enhancement.

### Autoimmunity and inflammation

Notably, EVs from different sources have also demonstrated tremendous potential in autoimmune and inflammation-related diseases, such as rheumatoid arthritis, psoriasis, and osteoporosis, exerting therapeutic effects through multiple mechanisms including remodeling the immune microenvironment, regulating inflammatory responses, and promoting tissue repair.

Mammalian EVs and PELNs are the candidates to be explored in the treatment of autoimmune and inflammation-related diseases. Mammalian EVs possess strong ability in regulating innate immune responses and tissue repair signals^[148]^. Recently, intestinal organoid-derived EVs are reported to regulate the gut-bone axis, further exerting therapeutic functions for osteoporosis^[149]^. PELNs, as edible nanocarriers, demonstrate increasingly evident therapeutic potential in this area^[150,151]^. A typical example is ginger-derived EVs (GDEVs), which are naturally rich in anti-inflammatory components such as 6-gingerol. Researchers engineered folate-modified GDEVs (FA-GDEVs) to enhance the targeting ability of GDEVs. Folate receptors are highly expressed on pro-inflammatory M1 macrophages within rheumatoid arthritis joints, enabling FA-GDEVs to actively target inflammatory sites. FA-GDEVs can effectively downregulate pro-inflammatory factors such as TNF-α and IL-6, thus significantly reducing joint swelling and bone erosion^[150,151]^. Despite advances in mammalian and plant-derived EVs, challenges still persist, prompting researchers to turn attention towards BEVs, which offer greater programmability and potential for mass production.

Probiotic-derived EVs, as postbiotics, exert their effects in rheumatoid arthritis through multiple pathways, including modulating immune cells, reducing pro-inflammatory factors, restoring intestinal barrier integrity, and inhibiting osteoclast activity^[152]^. For diseases requiring multi-stage sequential treatment, such as osteoporotic fractures (OPF), researchers have developed sophisticated BEV-based platforms that address the distinct phases of healing. Building on prior work demonstrating the feasibility of synthetic biology-based BEVs for bone-related conditions^[153]^, a recent breakthrough study used synthetic biology techniques to engineer bacteria that produce eBEVs simultaneously carrying bone morphogenetic protein-2 (BMP-2) and vascular endothelial growth factor (VEGF). Subsequently, the constructed BEVs carrying BMP‑2 and VEGF (BEVs‑BP)were co-encapsulated with IL-4 within a methacrylamide-modified gelatin (GelMA) hydrogel, forming IL-4/BEVs-BP@GelMA. This system enabled temporally controlled release: upon injection into the fracture site, the hydrogel rapidly released IL-4, swiftly ameliorating the inflammatory microenvironment at the lesion site. Then, the BEVs-BP were slowly released and, after internalization by surrounding cells, the secretion of BMP-2 and VEGF promoted osteogenic differentiation and angiogenesis^[154]^. As for the treatment of psoriasis, a novel strategy has been developed that consists of the large-scale production of *P. aeruginosa-* derived OMVs (Pg OMVs), and its further loading into thermosensitive PF-127 hydrogels. The subcutaneous injection of Pg OMVs hydrogels induces immunomodulatory effects that effectively suppresses skin inflammation and ameliorates psoriasis symptoms^[155]^. These engineering strategies demonstrate the significant potential of eBEVs in treating complex systemic diseases.

Therapies based on eBEVs hold promise for delivering safer, more effective and personalized treatment options for patients with autoimmune and inflammatory diseases, thereby genuinely driving transformative change in clinical treatment paradigms.

### Cancer therapy

In the field of cancer therapy, BEVs, particularly OMVs, have emerged as a highly promising next-generation smart nanomedicine platform. This is due to their innate immune-activating properties, exceptional tumor tissue enrichment capacity [the enhanced permeability and retention effect (EPR effect)], and high degree of engineering potential^[156]^. Other core advantages of the use of OMVs reside in the integration of multiple functions: targeted delivery, immune activation, and drug loading. Currently, eBEVs for cancer therapy primarily focus on four key strategies: surface functionalization and modification, therapeutic payload delivery, *in situ* construction of living factories, and regulation of the immune microenvironment.

Surface functionalization modifications aim to enhance the targeting efficiency and improve the safety profile of nBEVs, mainly through genetically engineered expression of fusion proteins, modification of target peptides, and fusion of hybrid membranes. eBEVs are uniquely positioned to address tumor heterogeneity. Their surface display platforms allow simultaneous presentation of multiple tumor-associated antigens, broadening the repertoire of targeted clones. For instance, researchers reported the use of genetic engineering techniques to express the vesicular stomatitis virus G protein on the surface of OMVs, enabling them to fuse with tumor cell membranes and achieve tumor heterogenization. These bacterial components can enhance the adjuvanticity and antigenicity of heterogenized tumor cells, effectively mobilizing the innate immune system to recognize and phagocytose tumors^[157]^. Furthermore, through techniques such as co-culture, electroporation, and chemical loading, therapeutic agents including chemotherapy drugs and nucleic acids can be encapsulated within BEVs, enabling the precise delivery of therapeutic payloads to tumor sites. Moreover, BEVs can serve as synthetic biologically-driven *in vivo* therapeutic factories, thus addressing the complexities of *ex vivo* production, purification, and drug loading processes. Sun *et al*. reported the administration of a genetically engineered *E. coli*, which produces high-yield OMVs loaded with therapeutic RNA, to mice via oral or injectable routes. The bacteria subsequently colonized tumors and continuously produced self-assembled OMVs loaded with PD-L1 siRNA within the tumor microenvironment, with relative RNA abundance 10^4^-10^7^ times higher than the mock group, leading to around 2-fold downregulation of PD-L1 gene expression and about 49.37% tumor suppression, achieving *in situ*, sustained drug production and delivery without complex *in vitro* procedures^[158]^. Notably, this “living factory” approach requires stringent biocontainment to prevent uncontrolled bacterial proliferation and horizontal gene transfer.

Lastly, numerous studies have directly eBEVs into cancer vaccines or immunomodulators, achieving regulation of the tumor immune microenvironment through mechanisms such as presenting tumor antigens and loading immunological adjuvants^[159]^. These distinct modification strategies are often employed in combination to enhance the application of BEVs in cancer therapy. A typical example consists of the genetic modification of OMVs derived from engineered low endotoxicity *E. coli* and functionalized with peptide angiopep-2 to obtain a nanoplatform capable of continuously traversing the blood-brain barrier (BBB) and targeting glioblastoma. Additionally, this platform is further loaded with doxorubicin and CD47 siRNA. Upon tumor targeting, the immunogenic components of the OMVs synergize with the encapsulated drugs to reprogram tumor-associated macrophages and microglia, effectively overcoming the immunosuppressive microenvironment of brain tumors^[160]^. eBEVs have also demonstrated remarkable synergy with immune checkpoint blockade therapy. One representative strategy involves genetically engineering OMVs to display the PD-1 ectodomain (OMV-PD1), which simultaneously activates systemic anti-tumor immunity via OMV’s intrinsic immunogenicity and blocks the PD-1/PD-L1 axis, resulting in superior tumor growth inhibition compared to either therapy alone^[96]^.

Compared to lipid or synthetic polymer nanoparticles, eBEVs possess intrinsic self-adjuvant properties due to their abundant pathogen-associated molecular patterns (PAMPs), which activate innate immunity without requiring external adjuvants, while offering a superior safety profile. Strain selection also determines the intrinsic immunogenicity of eBEVs. The LPS‑rich OMVs from *S. typhimurium* provide potent adjuvant effects, whereas the milder, LPS‑free CMVs from *Lactobacillus* deliver safer, low‑immunogenicity vehicles for targeted drug delivery. Taken together, eBEVs have evolved from simple bacterial nanostructures into functionally programmable antitumor agents through the convergence of synthetic biology and nanotechnology. Future developments will focus on multifunctional integration, precise controlled release, and addressing standardization and safety challenges in large-scale production and clinical translation. As research progresses, eBEVs hold promise to pioneer novel technical pathways for precision immunotherapy and combination cancer therapies.

### CNS disorders

CNS disorders, which affect the brain and spinal cord, constitute a complex and life-threatening category of diseases. Due to their progressive nature and limited treatment options, they present a significant global health challenge^[161]^. While the BBB maintains CNS stability, it also blocks over 98% of potential therapeutic compounds, presenting a major obstacle to effective treatment^[162]^. Developing effective therapies for CNS disorders requires overcoming two primary challenges: crossing the BBB and addressing complex underlying pathological mechanisms.

EVs from diverse sources, acting as nanoscale messengers, have been demonstrated to traverse the BBB through various pathways, making them a promising therapeutic avenue for treating CNS diseases^[163]^. Mesenchymal stem cell-derived exosomes (MSC-Exos) can enhance functional recovery in stroke rats while promoting angiogenesis, neurite remodeling and neurogenesis^[164]^. Also, MSC-Exos can reduce inflammation and inhibit abnormal neurogenesis on astrocytes^[165]^. PELNs, acting as neuroprotective agents, can modulate pathological processes such as neuroinflammation and oxidative stress by delivering special plant-derived bioactive molecules, including microRNAs, lipids and polyphenols^[166,167]^. Currently, an increasing number of studies are turning their attention to BEVs, owing to their close association with the gut-brain axis and their amenability to engineering modifications^[22]^.

BEVs can transmit crucial signals via the gut-brain axis^[168]^, thereby regulating neuroinflammation and neuropathic injury. *L. paracasei*-derived EVs show the ability to alleviate hyper-ammonemic rat models by reducing neuroinflammation, restoring GABA neurotransmission, and improving motor coordination by mediating “gut-brain axis” communication^[169]^. Moreover, BEVs may also improve cognitive deficits by regulating neurotrophic factors such as brain-derived neurotrophic factor (BDNF) and other proteins related to cognitive function^[170]^. However, research reports on the direct application of eBEVs in the treatment of CNS diseases remain exceedingly scarce. Nevertheless, portable engineering strategies from successful cases in other fields can be extracted and applied. Firstly, since the brain has a unique immune-privileged environment, and the surface of BEVs contains pathogen-associated molecular patterns such as LPS^[14]^, they may trigger strong neuroinflammatory responses, which inspires future studies to modify BEVs to achieve “reduced immunogenicity”. Secondly, neurotrophic factors (such as BDNF, GDNF) or siRNAs targeting Aβ/tau can be loaded into BEVs through synthetic biology or surface display targeting peptides.

In summary, the application of eBEVs in CNS disorders remains at the conceptual stage. Compared to other nanocarriers, eBEVs face the additional challenge of PAMP-induced neuroinflammation, as LPS or TA/LTA can trigger microglial activation and exacerbate CNS pathology. Moreover, the heterogeneous size of BEVs may affect their transport across the BBB. Achieving consistent BBB penetration while avoiding off-target brain inflammation remains a key hurdle for eBEV-based CNS therapies. Future breakthroughs may hinge on the combination of different approaches, for example, developing conditional penetration engineering strategies that intelligently respond to the microenvironment of brain lesions. As well, integrating materials science with synthetic biology to construct biomimetic hybrid eBEVs that minimize immunogenicity represent a promising direction.

### Skin regeneration

Beyond the aforementioned applications, EVs demonstrate significant potential in promoting tissue regeneration and repair due to their inherent biocompatibility, low immunogenicity, and exceptional capacity as carriers for bioactive molecules. EVs derived from mammalian cells have been extensively studied, with those originating from stem cells or functionally specialized cells demonstrating potent and comprehensive restorative capabilities^[171,172]^. PELNs naturally harbor active components capable of modulating the functions of diverse cell types, including stem cells and fibroblasts, thereby regulating cellular behavior and influencing key pathophysiological processes involved in wound healing^[173]^.

BEVs offer a novel and more cost-effective pathway in treating skin problems, with non-animal-derived ingredients aligning more closely with consumer expectations. nBEVs, especially those from probiotics, act by delivering intrinsically bioactive molecules such as miRNA. These molecules can promote epithelialization and angiogenesis^[174,175]^, while regulating macrophage mitochondrial function^[176]^, and ultimately facilitating wound repair. The engineering modification approaches primarily work at three core dimensions: functional component loading, exploration of new functional components, and delivery system optimization, which aim to overcome the therapeutic limitations of nBEVs.

Loading functional components represents the most direct enhancement strategy. For example, the incorporation of catalase and growth factors in BEVs can improve the oxidative stress microenvironment at the wound site and also accelerate tissue regeneration. A genetic engineering strategy that localizes superoxide dismutase (SOD) on *B. subtilis*-derived EVs could greatly enhance the stability and maintain the activity of SOD, and exhibits potential in restoring skin damage^[177]^. In addition to conventional loading, discovering and exploiting certain special functional molecules carried by BEVs also represents a promising strategy. For instance, it was found that BEVs derived from *Streptococcus mutans* (Sm EVs) can effectively promote skin healing, by carrying the transfer RNA (tRNA) fragments. By utilizing electroporation to load high amounts of tRNA isolated from Sm EVs into EcN EVs, it was found that the tRNA could activate the Toll-like Receptor 3 (TLR3) pathway, thereby promoting the proliferation and migration of epithelial cells and the healing of skin wounds^[178]^. Lastly, constructing multi-functional delivery systems, the fusion of *Lactobacillus* EVs into a photothermal hydrogel created an integrated platform “optical heating-oxygen release-BEVs therapy”. Near-infrared light irradiation can generate a mild thermal effect for antibacterial purposes and promote the release of BEVs. The oxygen released by BEVs synergistically regulates the phenotype of macrophages, achieving a complete set of responses that include antibacterial and anti-inflammatory effects to promote angiogenesis^[179]^. In another study, microjet technology was used to create artificial LCEV for skin rejuvenation and prevention of photoaging^[180]^. These examples all demonstrate the potential of eBEVs in the field of cosmetic skincare.

In conclusion, the engineering transformation has enabled BEVs to become programmable “intelligent nanorobots”. Through various engineering transformation strategies, eBEVs demonstrate significant potential to outperform their natural versions in addressing the complex pathological conditions of chronic wounds (such as infection, hypoxia, and inflammation).

Collectively, eBEVs have demonstrated remarkable therapeutic versatility across a broad spectrum of diseases, including intestinal disorders, metabolic diseases, autoimmune conditions, cancer, CNS disorders, and skin injuries. A systematic comparison of disease-specific engineering strategies, therapeutic outcomes, and advantages over non-BEV approaches is provided in Table 3. Despite the largely preclinical nature of current evidence, the rapid pace of innovation in synthetic biology and nanomedicine positions eBEVs as promising candidates for next-generation precision postbiotics, with several candidates poised for clinical translation in the coming years.

**Table 3:** Therapeutic applications of eBEVs in various diseases

**Types of diseases**	**Typical engineering strategies**	**Key therapeutic outcomes**	**Stage of development**	**Comparison with other approaches**
Intestinal diseases	Genetic engineering for yield enhancement; Surface display of antimicrobial peptide; Physical loading (ultrasound/extrusion)	Restoration of intestinal barrier, modulation of gut microbiota, alleviation of inflammation	Preclinical	Better targeting than conventional anti-inflammatory drugs; Higher safety than live probiotics
Metabolic disorders	Genetic engineering for self-assembly loading of therapeutic proteins; Microfluidic sonication loading	Reduction of uric acid, weight loss, amelioration of fatty liver	Preclinical	Higher resistance to gastric degradation than oral enzymes; Better biocompatibility than synthetic NPs
Autoimmunity and inflammation	Genetic engineering for co-expression of BMP-2/VEGF; Hydrogel co-encapsulation with IL-4; Thermosensitive hydrogel loading	Promotion of bone regeneration, alleviation of inflammation, modulation of macrophage polarization	Preclinical	Temporal synergy compared to monotherapy; Higher yield, lower cost than stem cell-derived EVs
Cancer	Surface display of targeting ligands; Loading of chemotherapeutics/nucleic acids; *In situ* “living factory” production	Anti-tumor immune activation, tumor regression, inhibition of metastasis	Preclinical	Endogenous adjuvanticity; Higher safety than viral vectors; Improved targeting than conventional chemotherapy
CNS disorders	Conceptual stage: BBB-crossing peptides (Angiopep-2); Detoxification (LPS modification); Loading of neurotrophic factors or siRNA	Still in proof-of-concept stage (BBB penetration and neuroprotective potential demonstrated)	Preclinical (concept)	Superior BBB penetration than conventional small molecules and nanocarriers
Skin regeneration	Surface display of SOD; Loading of tRNA; Fusion with photothermal hydrogel; Microfluidic artificial vesicles	Promotion of re-epithelialization, angiogenesis, antibacterial effects, anti-photoaging	Preclinical	Higher stability than growth factors; Superior to stem cell therapies (non-animal derived

eBEVs: Engineered bacterial extracellular vesicles; NPs: nanoparticles; BMP-2: bone morphogenetic protein-2; VEGF: vascular endothelial growth factor; IL-4: interleukin-4; EVs: extracellular vesicles; CNS: central nervous system; BBB: blood-brain barrier; LPS: lipopolysaccharide; siRNA: small interfering RNA; SOD: superoxide dismutase; tRNA: transfer RNA.

## CHALLENGES AND FUTURE PERSPECTIVES

Despite demonstrating significant potential in tackling different diseases, eBEVs still face a series of critical challenges in their transition from laboratory to clinical application^[181]^. Systematically understanding and overcoming these obstacles is essential to achieve their full therapeutic value.

### Standardized production of eBEVs

There is a crucial bottleneck concerning production, characterization, and standardization. Although bacteria possess the advantages of rapid proliferation, achieving efficient, stable, and reproducible large-scale production of BEVs remains the primary difficulty^[182]^. Current laboratory-scale preparation methods (such as ultracentrifugation) are difficult to scale up, and yield and purity of BEVs often cannot be achieved simultaneously. Moreover, different bacterial strains and cultivation conditions (such as stress states) significantly impact BEVs yield and composition, leading to batch-to-batch variability^[183]^. BEVs constitute a highly heterogeneous population, exhibiting considerable diversity in particle size, membrane composition, and internal cargo (proteins, nucleic acids, metabolites)^[184]^. The current lack of rapid, precise, and standardized characterization techniques to comprehensively evaluate their physical properties, biochemical composition, drug loading capacity, and functional activity poses significant challenges in establishing unified quality control standards^[185]^.

### Biosafety and immunogenicity of eBEVs

Another crucial challenge for the clinical use of eBEVs is the precise regulation of their biosafety and immunogenicity. BEVs from G-/G+ bacteria harbor PAMPs such as LPS and TA/LTA, respectively, on their surfaces, which act as potent immune stimulants^[16]^. This trait underpins their use as vaccine adjuvants or immunotherapy activators, it may also trigger uncontrolled systemic inflammatory responses, posing significant safety risks. Consequently, achieving detoxification through genetic engineering or chemical modification while preserving essential immunomodulatory functions represents a core challenge in the engineered design. Specific detoxification strategies have been developed, including lipid A engineering (e.g., knockout of *msbB*, *pagL*, *pagP*, or *lpxE*/*lpxF*/*futB*) and flagellin elimination, which can reduce endotoxicity by 10- to 25-fold while preserving adjuvant activity^[186,187]^. The balance between immunogenicity (desired for vaccines) and toxicity (undesired for drug carriers) is context-dependent and requires careful dose optimization. In Table 4, we provide a summary of balancing strategies across different application scenarios.

**Table 4:** A summary of balancing strategies across different application scenarios

**Application scenario**	**Desired immunogenicity**	**Acceptable toxicity level**	**Recommended engineering strategy**
Cancer vaccine	High (adjuvant effect needed)	Moderate (local inflammation acceptable)	Partial LPS attenuation (*msbB* knockout); Surface antigen display
Infectious disease vaccine	Moderate to high	Low (systemic safety priority)	Lipid A modification (*pagL*, *pagP*); Detoxification + alum co-administration
Targeted drug delivery	Low (avoid premature clearance)	Minimal	LPS-free OMVs; stealth coating (PEGylation)
Autoimmune disease therapy	Low (avoid immune activation)	Minimal	LPS-free OMVs; hybrid vesicles with inert carriers

LPS: Lipopolysaccharide; msbB: lipid A biosynthesis myristoyl transferase; pagL: lipid A 3-O-deacylase; pagP: lipid A palmitoyltransferase; OMVs: outer membrane vesicles; PEGylation: polyethylene glycol modification.

Preclinical safety assessment should encompass *in vitro* cytokine release assays, *in vivo* acute/chronic toxicity studies, and pyrogenicity testing. Furthermore, the *in vivo* fate of eBEVs remains incompletely elucidated. They may undergo rapid clearance by the mononuclear phagocyte system or accumulate in non-target tissues, triggering unintended effects^[181]^. Mitigation strategies include polyethylene glycol modification (PEGylation), targeting ligand conjugation, and biomimetic membrane hybridization. Lastly, comprehensive assessment of long-term biosafety is required, including whether BEVs derived from engineered bacteria could transfer genetic material such as antibiotic resistance genes at the systemic level^[185]^. Containment strategies, such as non-antibiotic selection markers, chromosomal integration, and suicide switches, can minimize this risk^[188]^.

Collectively, addressing these multifaceted safety and immunogenicity challenges requires an integrated approach combining rational genetic detoxification, rigorous preclinical assessment, advanced biodistribution tracking, and robust containment strategies. Such efforts will be essential to pave the way for the successful clinical translation of eBEVs.

### Future perspectives of eBEVs

Based on the challenges outlined above, several promising research directions are anticipated to accelerate the clinical translation of eBEVs. To overcome the production and standardization bottleneck, future efforts should focus on elucidating the molecular mechanisms of BEV biogenesis in different bacteria (particularly probiotics), which will provide theoretical guidance for rational engineering. High‑throughput screening platforms integrating synthetic biology, automation, and artificial intelligence can accelerate the optimization of hypervesiculating strains. Additionally, developing advanced characterization techniques, such as single‑particle analysis and multi‑omics profiling, will enable real‑time quality monitoring and standardization. Also, in response to the safety and immunogenicity concerns, future research should develop “conditional penetration” engineering strategies, such as stimulus‑responsive eBEVs (e.g., low pH, high ROS, specific enzymes) that activate them only in the disease microenvironment. Biomimetic hybrid vesicles that combine BEVs with carriers (e.g., liposomes or cell membranes) offer another avenue to reduce immunogenicity while preserving targeting capabilities. Furthermore, advanced *in vivo* imaging techniques should be employed to systematically map the biodistribution and clearance kinetics of eBEVs.

The regulatory classification of eBEVs varies across jurisdictions. In the United States, eBEVs are regulated as biological products of the Public Health Service Act by the Food and Drug Administration (FDA)’s Center for Biologics Evaluation and Research (CBER)^[189]^. In the European Union, they are likely classified as Advanced Therapy Medicinal Products (ATMPs), subject to centralized approval via EMA’s Committee for Advanced Therapies. For first-in-human trials, an Investigational New Drug (IND) application is required in the US, while a Clinical Trial Authorization (CTA) is needed in the Europe (EU), both demanding rigorous chemistry, manufacturing, and control (CMC) data as well as Good Manufacturing Practice (GMP) compliance^[182]^. Notably, eBEVs derived from genetically modified bacteria face additional regulatory complexity, including biosafety assessments and environmental risk evaluations under genetically modified organism (GMO) legislation. However, ongoing efforts - such as the proposed European Biotech Act - aim to streamline these requirements for ATMPs containing GMOs that pose minimal risk. To pave the way for regulatory and translational approval, international organizations should work toward establishing standardized quality control frameworks and regulatory guidelines tailored to eBEVs. Early engagement with regulatory agencies is encouraged to align development protocols with evolving expectations. Additionally, combining eBEVs with existing therapies (e.g., checkpoint inhibitors, chemotherapies) may accelerate clinical adoption by leveraging their synergistic effects.

In summary, the successful clinical translation of eBEVs will require an integrated, challenge-driven research agenda.

## CONCLUSION

In this review, we provide a state of the art scenario of BEVs utilization as postbiotics and directions for their engineered modification, efficacy, and safety. In recent years, EVs have emerged as key participants in cross-cell communication, regulating human health through the diverse bioactive molecules they carry. BEVs possess advantages such as rapid proliferation, abundant gene editing methods, and mature high-density culture techniques, garnering increasing attention, particularly when applied as postbiotics. This approach effectively circumvents various risks associated with injectable therapies, offering a safer and more effective means of improving human health. eBEVs enable the enhancement of their inherent functions, the conferral of novel capabilities, the mitigation of potential risks, increased yield, and improved production efficiency, all while preserving their naturally beneficial components, which facilitates flexible therapeutic strategies, better meeting the demands for health improvement. Emerging evidence indicates that the inherent properties of the parent bacterial strain, including Gram type, envelope structure, LPS/LTA content, basal yield, and cargo composition, can critically determine the baseline characteristics of eBEVs and should guide strain selection for specific therapeutic applications. Tailoring the engineering strategy to a carefully chosen strain thus represents the foundation for successful translation of eBEV‑based precision postbiotics. Looking forward, eBEVs embody the concept of “precision postbiotics” by integrating four engineering-enabled capabilities: targeted delivery, controlled release, programmable cargo loading, and detoxification. This precision framework transforms BEVs from naturally occurring but poorly controllable messengers into customizable therapeutic platforms.

## DECLARATIONS

### Copyright

© The Author(s) 2026.
